# Case report: A fatal outcome from co-infection of COVID-19 and dengue in the western region of Jeddah, Saudi Arabia

**DOI:** 10.3389/fpubh.2022.942381

**Published:** 2022-08-16

**Authors:** Ashwaq M. Al-Nazawi, Ali A. Al-Zahrani, Anjum Qadir, Rana Alghamdi, Ernest Tambo, Abdullah Alsahafi

**Affiliations:** ^1^Preventive Medicine Department, Public Health Directorate, Ministry of Health, Jeddah, Saudi Arabia; ^2^Health Holding Company, Jeddah, Saudi Arabia; ^3^Faculty of Health Sciences, Sydney Brenner Institute for Molecular Bioscience, Wits 21st Century Institute, University of the Witwatersrand, Johannesburg, South Africa

**Keywords:** Saudi Arabia, dengue (DENV), COVID-19, public health, co-infection

## Abstract

**Introduction:**

Co-infection of coronavirus disease 2019 (COVID-19) and dengue may coexist, as both viruses share similar laboratory and clinical features, making diagnosis and treatment challenging for health care professionals to prescribe, negatively impacting patient prognosis, and outcomes.

**Results and discussions:**

Both cases were positive for PCR and X-ray laboratory investigation at clinical examination, confirming COVID-19 and dengue co-infection, admission, and better management in referral hospitals are presented and discussed. The timeline provides detailed cases of situational analysis and the medical actions taken, as well as the outcomes.

**Conclusion:**

Both co-infection cases' (patients) health conditions had a poor prognosis and diagnosis and ended with undesired outcomes. Scaling up dual mosquito-vector linked viral diseases surveillance in understanding the transmission dynamics, early diagnosis, and the timely and safe monitoring of case management in clinical and hospital settings nationwide is paramount in curbing preventable co-infections and mortality.

## Introduction

As the world faces the COVID-19 pandemic with several resurgences of variants, the healthcare burden could be enormous where and when both COVID-19 and dengue co-infection coexist. SARS-CoV2, the virus causing the global pandemic, has been declared a Public Health Emergency of International Concern (PHEIC) by the WHO in early April 2020. COVID-19 has been recorded in more than 200 countries, with the emergence and spread of several COVID-19 virus variants and severity in different waves across nations (1). So far, 554.128 million confirmed COVID-19 cases have been recorded, and over 6.361 million deaths worldwide (1). A further complication of this pandemic arises in some tropical and subtropical countries as arbovirus outbreaks, such as dengue also exist. In tropical regions where arboviruses and COVID-19 may coexist, diagnosis is difficult as both viruses share similar laboratory and clinical features (2). Hence, testing for COVID-19 and dengue are necessary to evaluate and offset the increased morbidity of these co-infections. Dengue fever is a mosquito-borne disease that affects 129 tropical and subtropical countries, threatening a population of ~4 billion at risk (2). South America and Southeast Asia are entering the high-risk season of dengue outbreaks.

Scanty literature has previously reported seasonal dengue with high COVID-19 case viral load in Brazil, Colombia, Mexico, the Philippines, Malaysia, Singapore, Thailand, India, Indonesia, and Saudi Arabia (2–4). Thus far, dengue and SARS-CoV-2 viruses' co-infection in patients has been documented in Singapore, Thailand, India, Bangladesh, and France. In Singapore, two people (one man and one woman over the age of 50) were co-infected with both COVID-19 and the dengue virus. In Bangladesh, two co-infection cases were also reported (3). In Reunion Island (France), an 18-year-old male was reported to be co-infected with both COVID-19 and dengue (4). In India, a 60-year-old patient died from a co-infection of dengue and COVID-19 in April (5).

To the best of our knowledge, this is the first report that has been conducted on COVID-19 and dengue co-infection case fatality in the western region of Saudi Arabia (2). It is assumed that the probability of COVID-19 and dengue co-infection may rise during the peak dengue season. However, the difficulty in distinguishing between these two viral infections makes diagnosis and care delivery challenging and negatively impacts patient prognosis by healthcare professionals. 2020 on 2 March, the first cases of COVID-19 were reported in Saudi Arabia. The number of COVID-19 cases in KSA has now exceeded 300,000 and has spread across the country (6). At COVID-19 pandemic time, Jeddah is a city also facing an annual dengue fever outbreak since 1994. More than a thousand dengue cases have already been confirmed in 2020 (7), and patients with co-infection are likely to emerge based on concurrent transmission dynamics of both viral diseases in Saudi Arabia. Both case reports described COVID-19 and dengue co-infection, which represent a public health concern for the Saudi Ministry of Health and its populations.

## Case report 1

A 37-year-old non-Saudi man was referred from a local polyclinic to the secondary level hospital in Jeddah with a preliminary diagnosis of dengue fever. The patient had come to the polyclinic with complaints of fever, nausea, vomiting, body ache, dry cough, and abdominal pain for 5 days. His blood examination revealed he was having leucopenia (3,500 per cu. mm) and thrombocytopenia (109 × 103/mm^3^). His urine analysis revealed glycosuria and ketone-positive, and RBS was noted to be 293 mg/dl. The patient had a history of diabetes mellitus and was suspected to have dengue fever; for better management, he was referred to the secondary level hospital.

On presentation in the hospital, the patient was normotensive, febrile at 39°C, and his pulse was 90/min. Abdominal examination showed tender epigastrium and chest examination showed bilateral rhonchi. His chest X-ray revealed right-sided basal pneumonia. The patient was suspected of COVID-19, along with dengue, and was admitted to an isolation room. His nasopharyngeal swab was taken for COVID-19 testing, which came positive for RT-PCR. Simultaneously, the patient also came positive for dengue PCR, confirming the co-infection in the patient. Laboratory investigation at clinical admission/presentation is given in Table 1.

**Table 1:** Demographics, clinical and laboratory parameters, and indices of the two patients.

**Investigation**	**On admission**		
	**Case 1**	**Case 2**	
Gender	Male	Male	
Age	37 years	52 years	
Co-morbidity	Diabetes	No co-morbidity	
Fever	Yes	Yes	
Headache	No	No	
Body ache (Myalgia)	Yes	No	
Joint pain (Arthralgia)	No	No	
Sore throat	No	No	
Nausea	Yes	No	
SOB	No	Yes	
Vomiting	Yes	No	
Cough	Yes	Yes	
Skin rash	No	No	
Abdominal pain	Yes	No	
**Main laboratory findings of the two patients**			
**Investigation**	**Normal value**	**On admission**	
		**Case 1**	**Case 2**
Hemoglobin	13.8–17.2 g/dl	15.5	15.5
Total leucocyte count	4,500–11,000/mm^3^	3,760	4,500
Differential leucocyte count	40–80/20–40/2–10/1–6/0–1	60/32/5/3/0	76/16.1/4.2/0.5/1.8
N/L/M/E/B (%)			
Platelet	150,000–450,000/ul	99,000*	95,000*
Aspartate transaminase	<35 IU/L	34	55*
Alanine transaminase	29–33 IU/L	39*	39*
Creatinine in serum	0.6–1.2 mg/dL	0.7	1.15
Blood glucose random	90–110 mg /dl	184*	113.4
C-reactive protein	0–3 mg/L	4	>199*
Ferritin in serum	20–250 ng/mL	252	2,984*
D-dimer in plasma	<0.2 μg/mL	>200*	2.696*
Sodium in serum (mmol/l)	135–145 meq/L	157*	181*
Potassium in serum	3.5–5 meq/L	7.1*	6.8*
Chloride in serum (mmol/l)	98–107 mmol/l	108	107
Magnesium in serum (mg/dl)	1.6–2.3 mg/dl	1.03*	4.3*
Calcium in serum (mg/dl)	8.4–10.2 mg/dl	1.8*	7.6*
Albumin in serum (ms/dl)	34–50 ms/dl	30*	24*
Urine ketones	Positive	N/A	2+^*^
Prothrombin time (seconds)	<14 s	14	14.7
Troponin I (high sensitive)	Male: 11–12	30*	19,710*
	Female: 9–11Ng/L		

*Abnormal lab results recorded.

E, eosinophil; L, lymphocyte; M, monocyte; N, neutrophil; B, basophils.

Case 1 patient was treated as per the Ministry of Health (MOH) guidelines for COVID-19, which included antibiotics, anticoagulants, oxygen, corticosteroids, and immunomodulator (as per MOH protocol and clinical scenario) during his stay in hospital. His oxygen saturation was 97% on room air with no dyspnoea. The patient continued with medical treatment, but on the 9th day of his admission, he developed a disturbed level of consciousness, marked respiratory distress, increased blood glucose level, sugar and ketones in the urine, and metabolic acidosis and was shifted to ICU on high-flow oxygen, IV fluids, and IV insulin. CT brain was done, which showed a normal result.

Case 1 patient started desaturation and was connected to a non-invasive mechanical ventilator. At this point, his BP was 130/70 mmHg, tachycardia 140 b/min, and tachypnoea 50/min. His condition started deteriorating in the ICU with a Glasgow Coma Scale (GCS) scale of 10/15 and electrolyte Imbalance, hypernatremia, and hypokalaemia. On the 5th day of his ICU stay, he started showing signs of improvement. His level of consciousness improved, and his GCS was 13/15 on oxygen mask BP 120/70 mmHg, RR 30/min, O_2_ saturation 94%, temp 37.5, and on HIR infusion. While most of the parameters were showing improvement, there was a decrease in platelets, and it reached 20,400 on day 11 of intensive care unit (ICU) stay even after a transfusion of platelets (6 units of platelets given). On day 13 of his ICU stay, the patient's level of consciousness decreased and he was desaturated on a non-invasive mechanical ventilator, was intubated, and put on a mechanical ventilator. ABG showed severe metabolic and respiratory acidosis and high troponin, D dimer, and pro-BNP. The patient developed Brady-systole and did not respond to CPR as per ALS protocol and expired.

## Case report 2

A 52-year-old male patient with no significant past medical history was presented to the ER of a tertiary hospital in Jeddah, with complaints of cough, SOB, and fever. The patient had no vomiting or diarrhea and was preliminarily suspected of COVID-19 infection. Chest X-Ray showed increased broncho-vascular marking. The nasopharyngeal swab was taken, which was positive for COVID-19. The patient was admitted to the isolation room, and on examination, the patient was conscious, alert, and oriented vitally. Febrile (39) desaturating on room air was 89% and was put on nasal prongs at 4 L of 96%, tachypnoea RR 28. Electrocardiogram (ECG) and Trop I were normal (Table 1).

The patient (case 2) has started treatment on the MOH guidelines with antibiotics, antipyretics, and a nebulizer, along with gastrointestinal and deep-vein thrombosis prophylaxis. Due to the low platelet count, he was suspected of dengue fever, and his blood sample was sent for dengue analysis.

He could not maintain saturation, so he was shifted to ICU on 3rd day of his admission to the hospital and was connected to NRBM with oxygen at the rate of 10 L varying at 92–99%. He also developed bilateral basal crackles. Triple therapy consists of 2 antivirals and interferon. Lopinavir/Ritonavir, Ribavirin, and interferon ware administered. The patient continued to be tachypneic and febrile (37.8) and was encouraged for awake-prone positioning, while saturation increased temporarily but continued to be febrile (39). He could not maintain saturation on NRBM and shifted to non-invasive ventilation with Bilevel positive airway pressure (BiPAP)/high-flow canula. The patient failed to improve saturation despite being on a high-flow nasal cannula (FNNC) with 100% O_2_ and a flow of 60 L/min. The patient was intubated and connected, and mechanical ventilation was started as per acute respiratory distress syndrome protocol. To maintain saturation, the patient was completely sedated and paralyzed with muscle relaxants. CT pulmonary angiogram was done for the patient, was negative for pulmonary embolism, and showed ground-glass opacities concomitant with severe COVID-19 infection. His serology for dengue was positive. The patient developed AKI-acute kidney injury and was started on continuous chronic renal replacement therapy. The patient developed atrial fibrillation and was treated with an Amiodarone-loading dose/infusion for 24 h. On 1 July 2020, patient 2 became hypotensive and was started on vasopressors, but he showed a poor response and continued to be hypotensive and had a cardiac arrest on 4 July 2020.

**Figure 1:**
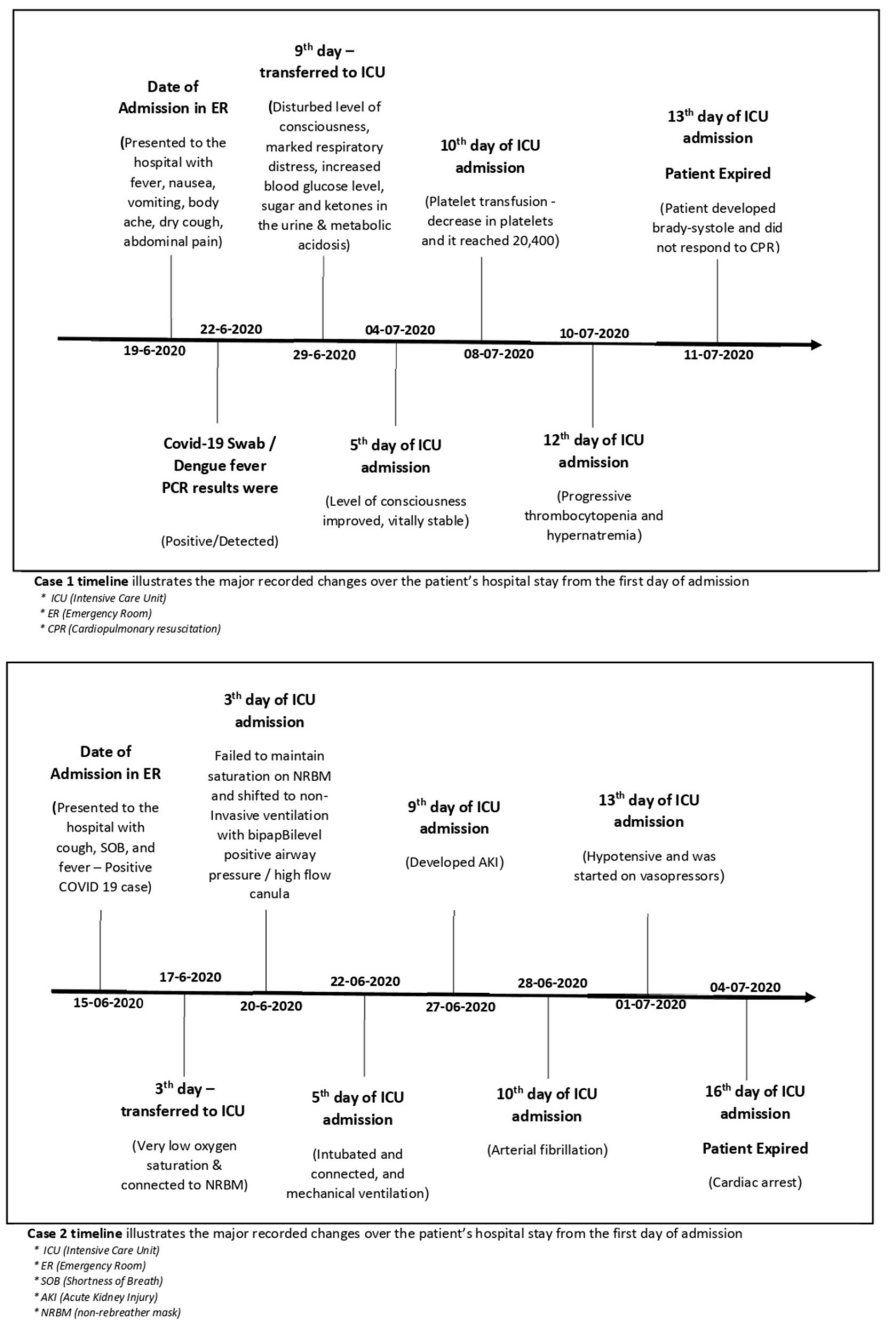
Summary of timeline and activities of both patients.

The timeline of both patients was presented and illustrated the care delivery, as well as the activities conducted from admission, hospitalization, and discharge (Figure 1).

## Discussion

We have presented our two patients who had co-infection of COVID-19 and dengue and had a fatal outcome. Interestingly, case 1 was preliminarily diagnosed as dengue fever, as his presentation was inclined more toward viral hemorrhagic fever, which included fever, body aches, nausea, and vomiting but turned out to be COVID-19-positive too; while case 2, whose presentation was more toward COVID-19 symptoms, which included fever, shortness of breath, and cough, was later found to be dengue-positive as well. Thrombocytopenia with leucopenia is a consistent feature of dengue fever. It has also been seen in COVID-19 infection. Both cases were presented with thrombocytopenia and leucopenia The similarity of this presentation in dengue and COVID-19 is one of the main challenges faced by health care workers during diagnosis and has been highlighted in many studies (4–6).

Lung involvement in COVID-19 infection is well-established and the chest X-ray for both cases was suggestive of pneumonia, showing the hallmark of lung involvement. Despite the symptomatic similarities between the two cases, the differences between the two were also evident in the laboratory investigation. In addition, case 1, which has a similar feature to case 2, was younger (35 years) than case 2 (52 years) but had diabetes mellitus as the risk factor. Nonetheless, both had a fatal outcome.

There were two death cases due to dengue fever that have been reported to the administration of vector-borne and zoonotic diseases in Jeddah. As we assessed the poor outcomes due to co-infection of both dengue and COVID-19, we focused on the reported death cases of dengue fever with positive COVID-19 tests, collected their files to study the prognosis, and highlighted the main change during their admission. We specifically chose them as they met the criteria of a narrow time frame between getting both diseases (<2 weeks). Moreover, we also assessed their previous dengue infection and their history of other infectious diseases and comorbidities.

Interestingly, we found out how both patients' health conditions were poorly prognosed and ended with undesired outcomes due to the co-infection. As we are reporting both death cases, the patients were compared in terms of their health condition during admission, and the significant change recorded in their labs/vitals, especially the platelet count since other studies shows that COVID-19 has an impact on platelets counts, as well as the dengue fever. Importantly, both patients did not receive COVID-19 vaccines and have been reported before the massive vaccination protocols applied by the MOH. As you have highlighted, this draft will be elaborated in a better and more precise way to clarify the importance of reporting fatal cases due to co-infection. Both cases' results are consistent with previous reports of dengue and COVID-19 co-infections burden and threats, which is still poorly assessed and reported consistently in most dengue-prone settings worldwide (8, 9).

## Conclusion and next steps

There is a major challenge in distinguishing diagnosis between COVID-19 and dengue co-infections that makes treatment challenging for healthcare professionals in Saudi Arabia. COVID-19 cases spread across the country now exceeded 796,268 confirmed cases and 9,211 deaths. More than a thousand dengue cases have already been confirmed in 2020, and the concurrent transmission dynamics of both viral diseases represent a public health concern for the Saudi Ministry of Health and its populations.

- Enhanced mosquito-vector and viral diseases surveillance, early diagnosis, and detection to safety and management monitoring are crucial in clinic and hospital settings in Jeddah and nationwide as well.
- Promoting dengue-effective and timely risk communication and scale of COVID-19 vaccination coverage, safety, and effectiveness metrics.
- Integrated implementation of COVID-19 and dengue community-based engagement, activities like health educational outreach in sustained preparedness, response, and recovery plans.

## Data availability statement

The original contributions presented in the study are included in the article/supplementary material, further inquiries can be directed to the corresponding author/s.

## Ethics statement

The ethical clearance was obtained from Jeddah Institute Review Board, IRB no. 1347, KCAST, KSA; H-02-J-002 dated 14/09/2020.

## Author contributions

AMA-N conceived and designed the study. AMA-N, RA, AQ, and AAA-Z drafted the manuscript. AAA-Z, RA, and AQ collated and analyzed data. AA obtained the patient files and coordinated with private and governmental health sectors. ET revised the primary draft. All authors have read and approved the final version of the manuscript.

## Funding

The project was supported by MOH, Jeddah, Saudi Arabia.

## Conflict of interest

AQ was employed by Health Holding Company. The remaining authors declare that the research was conducted in the absence of any commercial or financial relationships that could be construed as a potential conflict of interest.

## Publisher's note

All claims expressed in this article are solely those of the authors and do not necessarily represent those of their affiliated organizations, or those of the publisher, the editors and the reviewers. Any product that may be evaluated in this article, or claim that may be made by its manufacturer, is not guaranteed or endorsed by the publisher.

## Abbreviations

KSA: The kingdom of Saudi Arabia
RBS: random blood sugar
pt.: patient
SOB: shortness of breath
HIR: regular insulin
ABG: arterial blood gas
BNP: B-type natriuretic peptide
ALS: amyotrophic lateral sclerosis
NRBM: standard non-rebreathing mask
MOH: Ministry of Health.
